# Zanthoxylum nitidum extract attenuates BMP-2-induced inflammation and hyperpermeability

**DOI:** 10.1042/BSR20201098

**Published:** 2020-10-23

**Authors:** Tao Hu, Zhiwen Luo, Kai Li, Shanjin Wang, Desheng Wu

**Affiliations:** 1 Department of Spine Surgery, Shanghai East Hospital, Tongji University School of Medicine, China; 2 Department of Sports Medicine, Huashan Hospital, Fudan University, China; 3 Key Laboratory of Inorganic Coating Materials CAS, Shanghai Institute of Ceramics, Chinese Academy of Sciences, China

**Keywords:** BMP-2, hyperpermeability, inflammation, nuclear factor kappaB, Spinal fusion, Zanthoxylum nitidum

## Abstract

Bone morphogenetic protein-2 (BMP-2) is commonly applied in spinal surgery to augment spinal fusion. Nevertheless, its pro-inflammatory potential could induce dangerous side effects such as vascular hyper-permeability, posing the need for manners against this condition. The present study aims to investigate the protective effect of Zanthoxylum nitidum (ZN) on BMP-2-related hyperpermeability and inflammation on the human umbilical vein endothelial cells (HUVECs). The results revealed that, in a concentration-dependent manner, BMP-2 enhanced the production of pro-inflammatory cytokines, including interleukin (IL)-1α, IL-1β, and tumor necrosis factor-α, which were, however, suppressed by ZN. ZN inhibited BMP-2-induced inflammatory response by suppressing the phosphorylation of NF-κBp65 and IκB, and the abnormal nuclear translocation of p65. Moreover, the inhibited expression intercellular tight junction protein VE-cadherin and Occludin caused by BMP-2 was blocked by ZN. The hyper-permeability of HUVECs induced by BMP-2, as expressed as the higher fluorescent intensity of dextran, was also reversed by ZN. Overall, these findings demonstrated that ZN antagonized BMP-2-induced inflammation and hyperpermeability. It could be a therapeutic candidate for the treatment of BMP-2-induced side effects during spinal fusion.

## Introduction

As an effective osteoinductive cytokine [1], bone morphogenetic protein-2 (BMP-2) is commonly applied to promote osteogenesis clinically [2]. In 2002, the FDA approved the use of the recombinant human bone morphogenetic protein-2 (rhBMP-2) for anterior lumbar interbody fusion [3]. This technique has brought a leap forward for spinal fusion surgery with a 100% spinal fusion rate notably higher than other conventional osteogenic materials [4,5]. However, the follow-up shows that BMP-2 can induce inflammation-related complications, including severe edema in the surgical area, increased postoperative drainage, and even the swelling of the soft tissue in front of the cervical vertebra that may compress the airway [6–9]. Therefore, searching for potential agents against inflammation caused by BMP-2 is of considerable significance to guarantee the safety of spinal fusion with BMP-2.

Apart from osteoblasts, local vascular endothelial cells are also regulated by BMP-2. By activating the NF-κB signaling pathway, BMP-2 induces the inflammatory response of human umbilical vein endothelial cells (HUVECs) with the production of various inflammatory cytokines [10–13]. Meanwhile, the down-regulation of VE-Cadherin and Occludin by BMP-2 leads to elevated vascular endothelial permeability, causing tissue swelling [14,15].

Currently, the anti-inflammatory drugs used to treat BMP-2 related side-effects include methylprednisolone and corticosteroids [16], the osteoinductive growth factor Nel-like protein 1 (NELL-1) [17], and rapamycin [18]. However, some terrible complications limit their clinical use [19,20]. For example, high dosage methylprednisolone treatment for patients with a cervical spinal injury may cause pulmonary side effects [19]. Therefore, researches are required for finding out better adjuvant drugs for BMP-2 treatment in the clinic.

Zanthoxylum nitidum (ZN), a Traditional Chinese Medicine also named Liang Mianzhen in Chinese, is a natural herb in Chinese folk medicine that has been applied in numerous disorders, including rheumatic conditions, stomach pain, arthralgia, and so firth since antiquity [21,22]. Recently, Qin also reported that the extract of ZN relieved CFA-induced inflammatory pain in a rat model [23]. Particularly, ZN has been commonly added in the toothpaste to reduce the inflammation in the oral cavity for more than 30 years, and its side effect has not been reported up to now. Hence, its safety is relatively warranted [23,24]. On the ground of this, we, in the present study, planned to investigate the ability of ZN in alleviating BMP-2-related inflammation in HUVECs, and whether it could reverse hyper-permeability.

## Materials and methods

### Cell culture

Human umbilical vein endothelial cells (HUVECs) were purchased from the American Type Culture Collection. They were grown in Endothelial Cell Medium (ECM) with high glucose supplied with 10% fetal bovine serum and 1% penicillin and streptomycin at 37°C in a humidified cell incubator with a 5% CO_2_ condition. When the whole-cell confluency reached about 80%, interventions were conducted.

### Intervention

rhBMP-2 was applied to HUVECs with concentration gradients (0, 10, and 20 ng/ml) for 8 h. ZN was purchased from Xi'an Qingzhi Bio-Tech Co., Ltd. (Xi’an, China). Three dosages (1, 10, and 20 μg/ml) were administered to HUVECs 4 h before 20 ng/ml rhBMP-2 intervention. At the end of interventions, cells were harvested for measuring mRNA and protein expression, while the supernatant was subject to the quantification of cytokines. HUVECs without any interventions served as the control group.

### Real-time qPCR (RT-qPCR)

RNA was extracted and analyzed using the previous method [25]. Total RNA was obtained by the Trizol reagent (Invitrogen, Carlsbad, CA) and quantified by Nanodrop. RNA was then reversely transcribed by the PrimeScript RT reagent kit (Takara Bio). Specific primers used in the experiment are from PrimerBank (Table 1). The experiment was performed on the ABI7900 Real-Time PCR System (Applied Biosystems). The expression of mRNAs relative to the expression of GAPDH was calculated and normalized to the control group.

**Table 1:** Primers used in the experiment

Genes	Primers (5′-3′)
IL-1β	F: CTTATTACAGTGGCAATGAGGATG
	R: CTTTCAACACGCAGGACAGGTACA
IL-1α	F: CGCCAATGACTCAGAGGAAGA
	R: AGGGCGTCATTCAGGATGAA
TNF-α	F: CCCGAGTGACAAGAATGTAG
	R: TGAGGTACAGGCCCTCTGAT
GAPGH	F: CAGGGCTGCTTTTAACTCTGGT
	R: GATTTTGGAGGGATCTGGCT

### Enzyme-linked immunosorbent assay (ELISA)

ELISA kits, including IL-1α, IL-1β, and TNF-α, were purchased from Laizee (LEH011-2, LEH012-2, LEH810-2). Cell supernatant of each group was collected, and then those kits were used according to the manufacturer’s instructions.

### Western blot analysis (WB)

Protein was extracted and analyzed using an established method [26]. In brief, the total protein from HUVECs was collected by RIPA lysis buffer (R0010; Solarbio, Beijing, China) containing Phenylmethanesulfonyl fluoride (PMSF; Solarbio, Beijing, China). Use the BCA Protein Assay Kit (Beyotime Biotechnology, Shanghai, China) to measure the concentration of protein. About 10 μg protein samples from each group were separated by 10% SDS-PAGE. Then, they transferred to nitrocellulose membranes. Utilize 5% non-fat milk dissolved in Tris-buffered saline containing Tween-20 to block the blots before applying primary antibodies overnight at 4°C. Anti-VE-Cadherin, anti-P-VE-Cadherin, anti-Occludin, anti-p65, anti-p-p65, anti-IκB, anti-p-IκB, and anti-GAPDH (Affinity) antibodies were used as primary antibodies (Table 2).

**Table 2:** Primary antibodies used in the experiment

Antibody	Source	Catalog No.	Type	Dilution	MW (kD)
NF-kB p65	Affinity	AF5006	Rabbit mAb	1:1000(WB)	65
				1:50(IF)	
p-p65 (Ser536)	Affinity	AF2006	Rabbit mAb	1:1000(WB)	65
IKB	Affinity	AF6014	Rabbit mAb	1:1000(WB)	85
p-IKB (Ser32/Ser36)	Affinity	AF3013	Rabbit mAb	1:1000(WB)	85
VE-Cadherin	Affinity	AF6265	Rabbit mAb	1:1000(WB)	120
				1:50(IF)	
p-VE-Cadherin (Tyr731)	Affinity	AF3265	Rabbit mAb	1:1000(WB)	130
Occludin	Affinity	DF7504	Rabbit mAb	1:1000(WB)	65
GAPDH	Affinity	AF7021	Rabbit mAb	1:1000(WB)	36

### Immunofluorescence (IF)

To examine the expression and location of p65 and VE-Cadherin, Immunofluorescence staining on HUVECs was conducted as previously described [27]. Briefly, cells were fixed with 4% paraformaldehyde about 8 h and then blocked with donkey serum for 30 min. After that, they were incubated with primary antibodies overnight at 4°C following incubation with matched second antibodies for 60 min at room temperature. Primary antibodies used were anti-p65 and anti-VE-Cadherin. DAPI was used to locate nuclei. Images were observed by fluorescence microscopy with ImageJ software for quantification.

For the measurement of the level of p65 and Occludin, the total expression was obtained from 5 high-power random fields. It was divided by the number of DAPI to acquire average expression. Then, the average expression was normalized to the control group. For comparing the difference of p65 location, the percentage of cells with p65 in nuclei was calculated and normalized to the control group.

### Cell counting kit-8 for cell viability (CCK-8)

The viability of HUVECs subjected to ZN was assessed with the CCK-8 assay according to the manufacturer’s direction (Nanjing KeyGen Biotech) [28]. HUVECs (2500/well) were seeded in 48-well plates with six replicates per group and cultured in 100 μl different concentrations of ZN (1, 10, 20, 50, 100 μg/ml) or serum-free DMEM for 8 h. CCK-8 solution (10 ml) was added to each well and incubated or 2 h at 37°C. Then, the absorbance at 450 nm of the supernatant was measured by a microplate reader (Victor X; PerkinElmer) (*n* = 6 per group)

### Cell permeability analysis

Permeability *in vitro* was carried out using transwell plates. HUVECs were seeded in the upper chamber with 200 μl of ECM, while the lower chamber was filled with 500 μl of ECM. When the plate achieved 100% monolayer cell coverage, various intervention combinations of the prementioned were conducted. Then, FITC-dextran (1 mg/ml) was incubated with the cells for 1 h, and the fluorescence of the medium in the lower chamber medium was evaluated by a microplate reader (Biotech, highland park, USA) at 494 nm excitation and 521 nm emission. The value of each group was normalized to the control group.

### Statistical analysis

All experiments were conducted in triplicate. Data, as expressed by mean ± SD, were analyzed with GraphPad Prism 7.0. Significance was checked by one-way ANOVA followed by the post hoc LSD test. *P*<0.05 was regarded as significant.

## Results

### BMP-2 increased inflammatory cytokines expression of HUVECs

**Figure 1:**
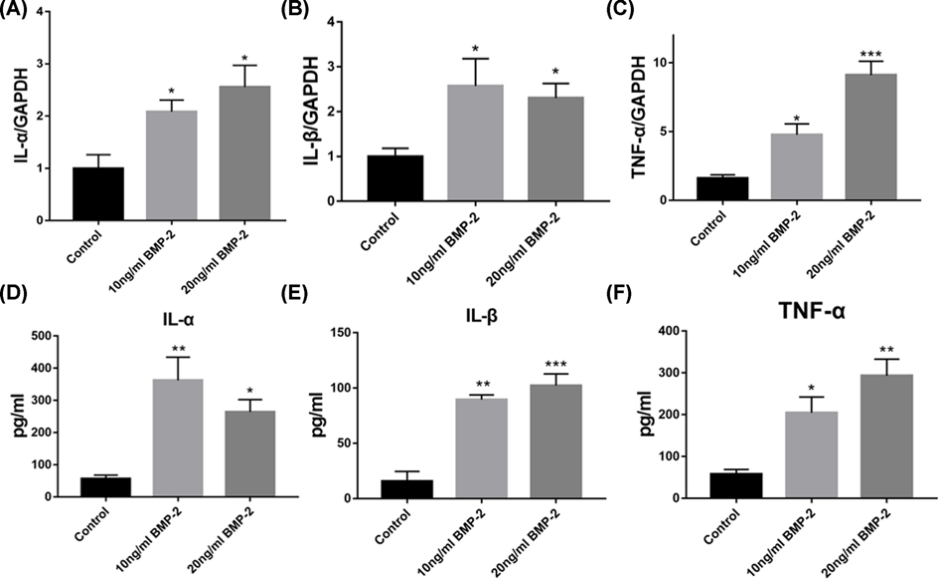
BMP-2 increased inflammatory cytokines expression of HUVECs (**A–C**) the gene expressions of IL-1α, IL-1β, and TNF-α in the HUVECs were investigated by qRT-PCR (*n* = 3–5). (**D–F**) the protein expressions of IL-1α, IL-1β, and TNF-α in the cell supernatant were investigated by ELISA (*n* = 3–5). Data are expressed as the means ± standard error of the mean; * *P*<0.05, **P<0.01, ***P<0.001; BMP-2, Bone morphogenetic protein-2; IL, interleukin; TNF, tumor necrosis factor; GAPDH, reduced glyceraldehyde-phosphate dehydrogenase.

To assess the inflammatory effects of BMP-2, the expressions of different cytokines were investigated by using both qRT-PCR and ELISA. Compared with the control group, the gene expression of IL-1α, IL-1β, and TNF-α increased significantly in the HUVECs after BMP-2 treatment for 1 d. Meanwhile, the levels of each gene increased following the increased concentration of BMP-2 (from 0 to 20 ng/ml), except IL-1β, which did not change notably between 10 and 20 ng/ml BMP-2 treatment group (Figure 1A–C).

ELISA kits elucidated IL-1α, IL-1β, and TNF-α protein expression in HUVECs supernatant. Compared with the control group, the protein expression of those inflammatory factors increased notably in HUVECs supernatant after 1d BMP-2 treatment (10 and 20 ng/ml), which was generally matched with their gene expression (Figure 1D–F).

### BMP-2 promoted the NF-κB signal pathway in HUVECs

**Figure 2:**
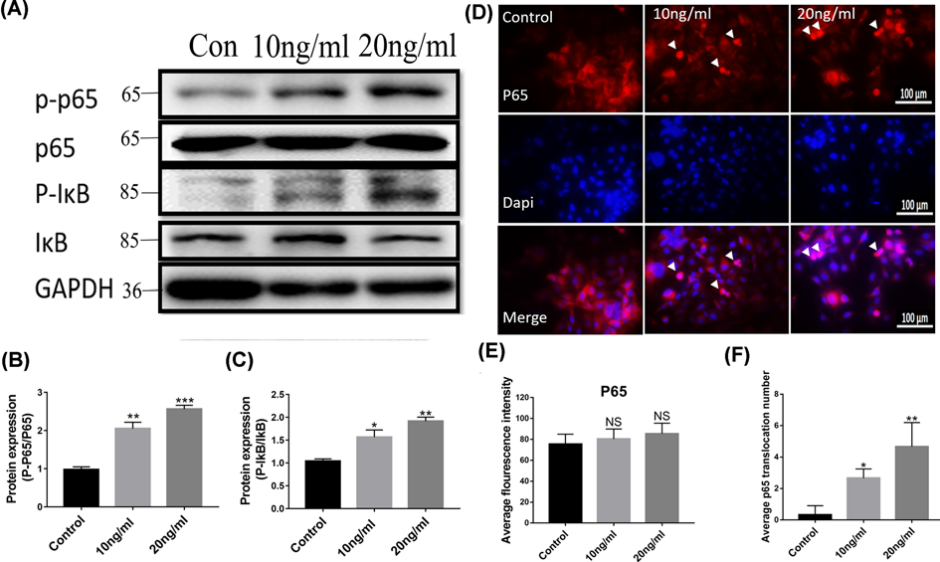
BMP-2 promoted the NF-κB signal pathway in HUVECs (**A–C**) p-IκB and p-p65 protein levels in HUVECs were determined (*n*=5). (**D**) Immunofluorescence localization and relative expression of NF-κBp65 in HUVECs. All photographs were taken at 40× magnification. (**E** and **F**) The average fluorescence intensity and nuclear translocation of p65 for each microscopic field were assessed. Data are expressed as the means ± standard error of the mean. * *P*<0.05, ** *P*<0.01, *** *P*<0.001; BMP-2, Bone morphogenetic protein-2; NF-κB, nuclear factor kappa-B; IκB, inhibitor of NF-κB; GAPDH, reduced glyceraldehyde-phosphate dehydrogenase.

To further investigate the inflammatory effects of BMP-2, the protein expressions of the NF-κB pathway were elucidated by Western blot analysis and immunofluorescence. We evaluated the phosphorylation of NF-κBp65 and IκB protein expression levels. We found that compared with the control group, both were up-regulated significantly in HUVECs after 10 and 20 ng/ml BMP-2 treatment for 1d, while total p65 and IκB protein expression did not change obviously (Figure 2A–C).

As shown in the left immunohistochemical staining picture, the fluorescence intensity of NF-κBp65 matched well with the Western blotting results. The staining results showed the expression of p65 notably increased in the nucleus of HUVECs after the two-doses treatment of BMP-2 for 1d, which indicated that BMP-2 induced the abnormal nuclear translocation of p65 (Figure 2D–F). The average p65 fluorescence intensity of each microscopic field was not significantly changed after BMP-2 treatment (Figure 2E).

### BMP-2 increased the HUVECs permeability

**Figure 3:**
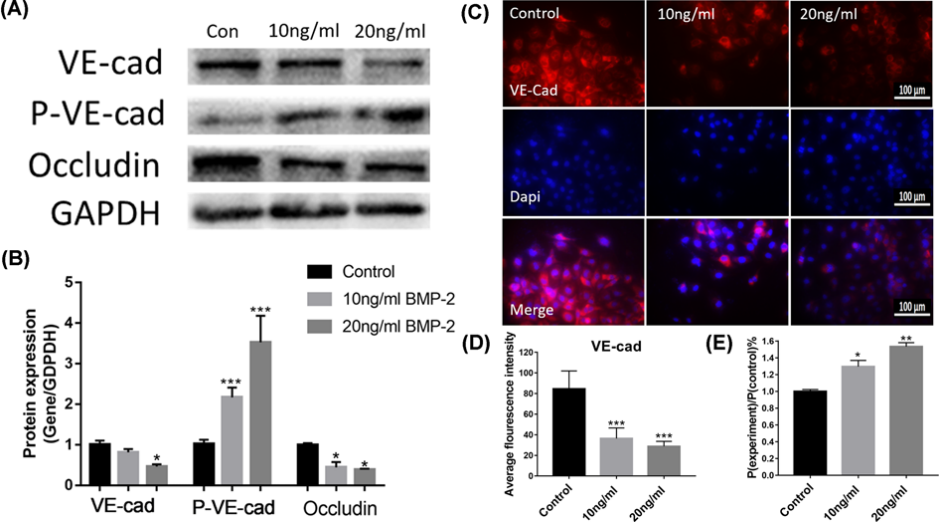
BMP-2 increased the HUVECs permeability (**A** and **B**) VE-cad, p-VE-cad, and Occludin protein levels in HUVECs were determined (*n*=5). (**C**) Immunofluorescence localization and relative expression of VE-cad in HUVECs. All photographs were taken at 40× magnification. (**D**) The average fluorescence intensity of VE-cad for each microscopic field was counted. HUVECs were planted on trans-well plates with a complete medium to achieve 100% monolayer cell coverage. Then, the complete medium in the upper chamber was replaced by medium containing 1 mg/ml FITC-dextran and BMP-2 was used to stimulate those cells. The cells were cultured in the cell incubator for 2 h. (**E**) The permeable fluorescent intensity of dextran of the monolayer HUVECs was determined by Trans-well and FITC-Dextran experiments. Data are expressed as the means ± standard error of the mean; * *P*<0.05, ** *P*<0.01, *** *P*<0.001 (compared with control group).

To investigate the effects of BMP-2 on the permeability of HUVECs, the protein expressions of VE-cad, p-VE-cad, and Occludin were elucidated by Western blot analysis and Immunofluorescence, and the permeability was directly assessed by Trans-well and FITC-Dextran. The expression of VE-cadherin down-regulated significantly in high concentration BMP-2 (20 ng/ml), while the moderate-dose group shown a slight downward trend (Figure 3A–C). In particular, the immunohistochemical staining showed the VE-cad present in the surrounding of the HUVECs. At the same time, its expression decreased obviously, which match the results of Western blot (Figure 3C,D). Meanwhile, the expression of Occludin presented a considerable decrease with an upward concentration of BMP-2. In contrast, the p-VE-cad protein expression was significantly up-regulated in HUVECs after 1d BMP-2 treatment (10 and 20ng/ml) (Figure 3B).

The permeable fluorescent intensity of dextran, compared with the control group, significantly increased approximately 24 and 48 percentages respectively, when the monolayer HUVECs were treated with 10 and 20 ng/ml BMP-2 (Figure 3E).

### The inhibitory effect of ZN on HUVECs viability

**Figure 4:**
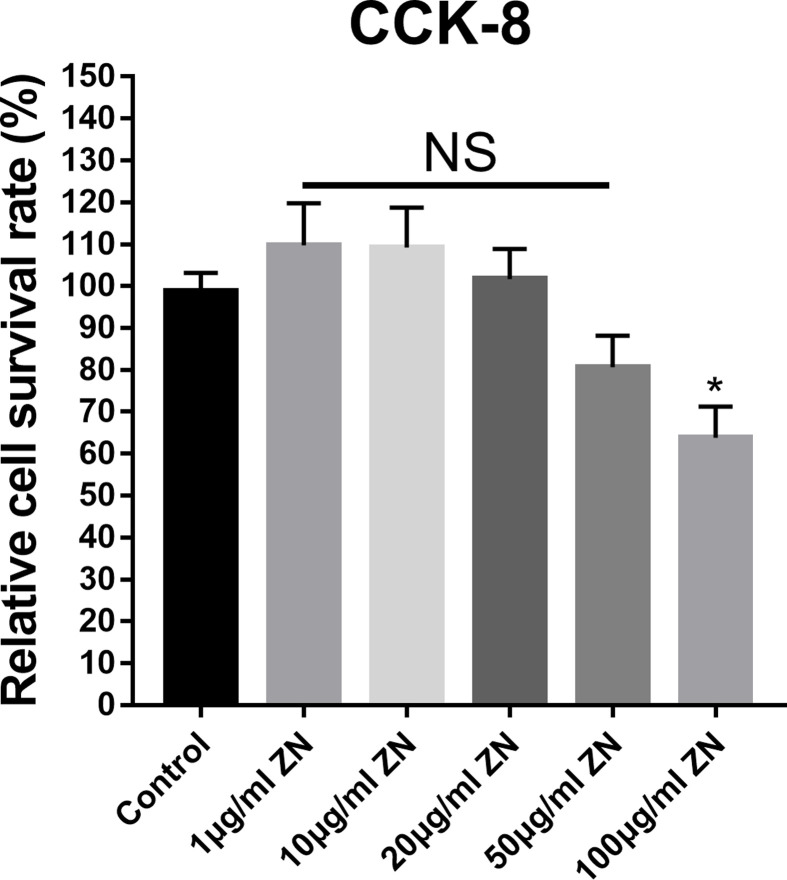
The inhibitory effect of ZN on HUVECs viability HUVECs were treated with the indicated doses of ZN for 8 h. The cell viability was measured by CCK-8 assay. Data are expressed as the means ± standard error of the mean.

According to the CCK-8 assay, the relative cell survival rate of HUEVCs in the 100 μg/ml ZN group for 8 h was 63.775%, which was significantly lower than that in the control group. In contrast, the rates in the other ZN treated groups (1, 10, 20, and 50 μg/ml) were not remarkably changed (Figure 4).

### Effect of ZN on inflammatory cytokines in BMP-2-induced inflammation of HUVECs

**Figure 5:**
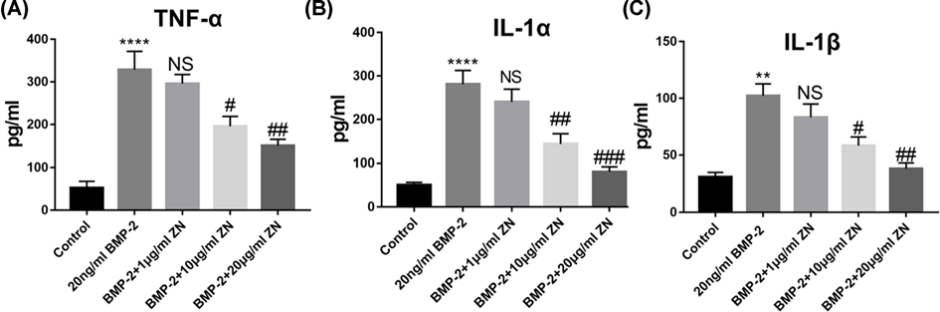
Effect of ZN on inflammatory cytokines in BMP-2-induced inflammation of HUVECs First, HUVECS were pre-treated with ZN (0, 1, 10, and 20 μg/ml) for 4 h. Then, cells were treated with BMP-2 (0, 10, and 20 ng/ml) for 8 h. (**A–C**) The protein expressions of IL-1α, IL-1β, and TNF-α in the cell supernatant were investigated by ELISA (*n*=3–5). Data are expressed as the means ± standard error of the mean; ** *P*<0.01, **** *P*<0.0001, ^#^*P*<0.05, ^##^*P*<0.01, ^###^*P*<0.001 (compared with BMP-2 group); ZN, Zanthoxylum nitidum.

To investigate the anti-inflammatory effects of ZN, the expressions of various cytokines were evaluated using ELISA. Compared with the control group, the expression of IL-1β, and TNF-α in the supernatant of HUVECs was significantly increased in the 20 ng/ml BMP-2 treatment group (1d) (Figure 5A–C). However, when the HUVECs were treated with moderate and high concentration ZN (10 and 20 μg/ml), their expression in the supernatant significantly decreased (Figure 5A-C).

### Effect of ZN on the NF-κB pathway in BMP-2-induced inflammation of HUVECs

**Figure 6:**
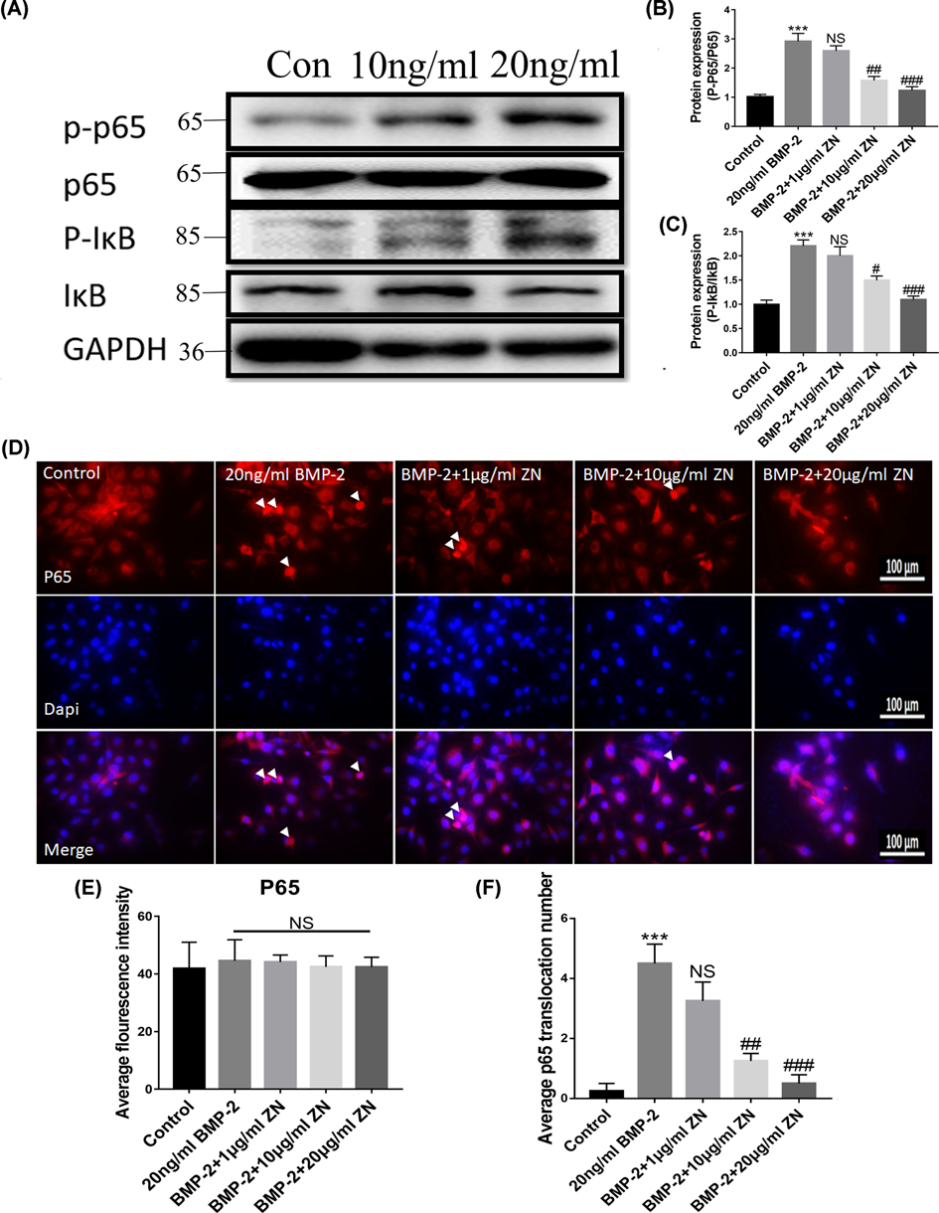
Effect of ZN on the NF-κB pathway in BMP-2-induced inflammation of HUVECs Cells were administered with BMP-2 (0, 20 ng/ml) and ZN (0, 1, 10, and 20 μg/ml). (**A–C**) p-IκB and p-p65 protein levels in HUVECs were determined (*n*=5). (**D**) Immunofluorescence localization and relative expression of NF-κBp65 in HUVECs. All photographs were taken at 40× magnification. (**E** and **F**) The average fluorescence intensity and nuclear translocation of p65 for each microscopic field were assessed. Data are expressed as the means ± standard error of the mean; *** *P*<0.001 (compared with control group); ^#^*P*<0.05, ^##^*P*<0.01, ^###^*P*<0.001 (compared with BMP-2 group).

To further investigate the anti-inflammatory effects of ZN, the protein expressions of the NF-κB pathway were assessed by Western blot and immunofluorescence. The phosphorylation levels of NF-κBp65 and IκB protein expression in the HUVECs were significantly higher than those in the control group (Figure 6A–C). However, when the HUVECs were treated with moderate and high concentration ZN, the phosphorylation levels of NF-κBp65 and IκB protein expression significantly decreased. In the low concentration ZN treatment group (1 μg/ml), the anti-inflammation effects were not statistically significant, but a downward trend was presented (Figure 6B,C). As the immunohistochemical staining has shown, the BMP-2-induced abnormal nuclear translocation of p65 was relieved with the increasing concentration of ZN treatment (0–20 μg/ml). In the high BMP-2 dosage group, there was no visible and positive p65 fluorescence presenting in the nucleus of the HUVECs (Figure 6D–F). The average p65 fluorescence intensity of each microscopic field was not significantly changed after BMP-2 and ZN treatment (Figure 6E).

### Effect of ZN on BMP-2-caused decreased HUVECs permeability

**Figure 7:**
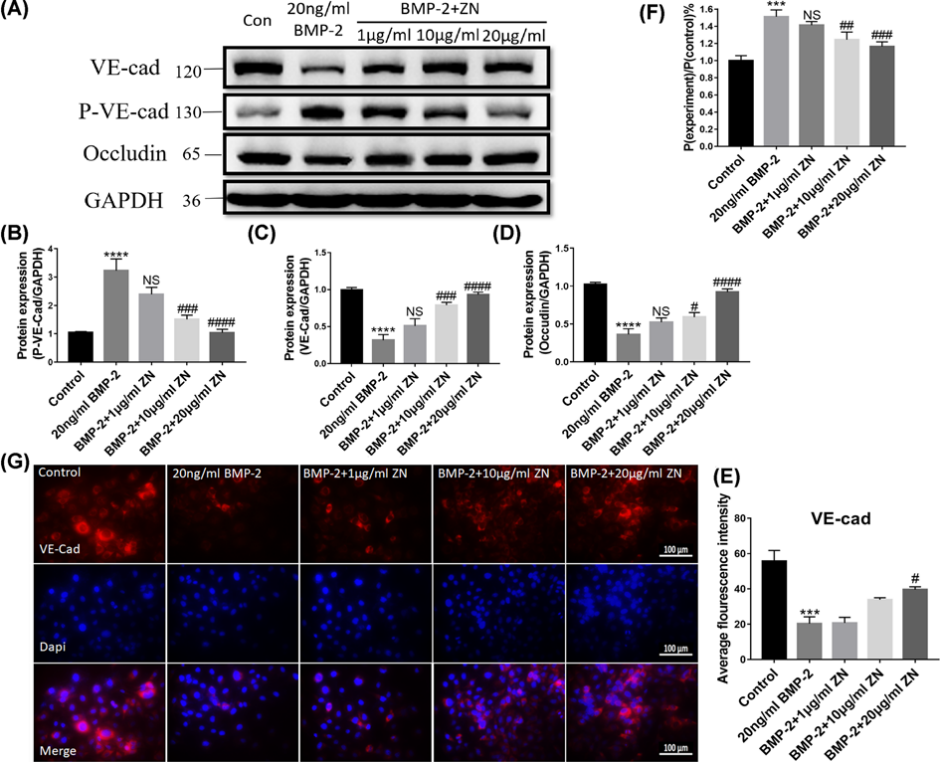
Effect of ZN on BMP-2-caused decreased HUVECs permeability Cells were administered with BMP-2 (0 and 20 ng/ml) and ZN (0, 1, 10, and 20 μg/ml). (**A–D**) VE-cad, p-VE-cad, and Occludin protein levels in HUVECs were determined (*n*=5). (**E**) The average fluorescence intensity of VE-cad for each microscopic field was counted. HUVECs were planted on trans-well plates with a complete medium to achieve 100% monolayer cell coverage. Then, the complete medium in the upper chamber was replaced by medium containing 1 mg/ml FITC-dextran, BMP-2, and ZN were used to stimulate those cells. The cells were cultured in the cell incubator for 2 h. (**F**) The permeable fluorescent intensity of dextran of the monolayer HUVECs was determined by Trans-well and FITC-Dextran experiments. (**G**) Immunofluorescence localization and relative expression of VE-cad in HUVECs. All photographs were taken at 40× magnification. Data are expressed as the means ± standard error of the mean. *** *P*<0.001, **** *P*<0.0001 (compared with control group); ^#^*P*<0.05, ^##^ *P*<0.01, ^###^*P*<0.001, ^####^*P*<0.0001 (compared with BMP-2 group); ZN, Zanthoxylum nitidum.

To further explore the effects of ZN on BMP-2-induced higher HUVECs permeability, the protein expressions of VE-cad, p-VE-cad, and Occludin were studied by WB/IF, and the permeability was directly analyzed by Trans-well and FITC-Dextran. The protein expression levels of VE-cad and Occludin sharply decreased after high concentration BMP-2 (20 ng/ml) treatment. In contrast, after a series of concentrations of ZN (1–20 μg/ml) treatment, their expressions were rescued gradually (Figure 7A–D). At the same time, compared with the control group, the protein expression levels of pVE-cadherin and the permeable fluorescent intensity of dextran in the HUVECs treated with BMP-2 significantly increased; in contrast, after the treatment of ZN, their levels presented a progressively downward trend in an increasing concentration manner (Figure 7A,B,F). Notably, the permeable fluorescent intensity of dextran of the 20 μg/ml ZN-treated group almost recovered to the level of the control group, which showed that ZN had a significant effect on the reduction of BMP-2-induced increased HUVECs permeability (Figure 7F).

As for the immunohistochemical staining, the protein expression of VE-cad surrounding the HUVECs was rescued by ZN treatment from the low expression level cause by 20 ng/ml BMP-2 (Figure 7E,G).

## Discussion

To the best of our knowledge, this is the first study to comprehensively investigate the antagonistic effects of ZN on BMP-2-induced inflammation and hyperpermeability in HUVECs. Our results indicated that ZN attenuated BMP-2 related inflammation with suppression of the NF-κB pathway and protected the permeability by restoring the expression of intercellular tight junction proteins.

The side effects of BMP-2 constitute a significant concern limiting its clinical use. Our previous study reported the cases of long segmental spinal orthopedic fusion with BMP-2, of whom the postoperative drainage volume of cases was significantly higher than that of cases without BMP-2 [29]. Robin et al. reported that patients who used BMP-2 in cervical fusion surgery were forced to undergo surgery for massive inflammatory exudates and swelling of the neck twice after an operation. Meanwhile, inflammatory factors such as TNF- α, IL-1, IL-6, and IL-8 were detected in the inflammatory exudates [30]. Similarly, we found that BMP-2 provoked the inflammatory reaction of HUVECs by exacerbating pro-inflammatory cytokines and NF-κB signaling pathway. As a typical response toward inflammation, increased vascular permeability is caused by down-regulating VE-cad and Occludin leading to the inflammatory exudation [14,15,29,31,32]. How to promote spinal fusion safely under the condition of controllable inflammation is an urgent problem to be solved.

At present, many methods have been developed to reduce the inflammatory side effects of BMP, such as the sustained release of BMP-2 [33], adjunctive corticosteroids along with BMP-2 [16,34], rapamycin [18,35], and so forth. However, no study has researched if ZN can alleviate the inflammatory side effects of BMP. Zanthoxylum nitidum has been identified as a useful drug to treat a wide range of diseases in recent years. Most recently, Lu et al. reported that ZN could be used to treat *Helicobacter pylori*-associated gastric diseases by inactivating urease in a concentration-dependent manner [36]. Additionally, a continually increasing number of researches have revealed the various functions of ZN in different kinds of diseases, including antiviral and antifungal effects [37], anti-inflammatory effects [38], and anticancer effects [39,40]. Thus, we investigated the effects and the underlying mechanism of ZN on BMP-2-induced inflammation/hyperpermeability.

In the present study, we found that ZN can significantly reduce BMP-2-induced inflammatory responses. Most directly, the activation of the NF-κB signal pathway and abnormal nuclear translocation of p65 caused by BMP-2 was blocked by ZN treatment. In the study of Qin *et al.*, they also demonstrated the anti-inflammatory effects of ZN might be through ERK and NF-κB signaling [23]. Besides, the inhibited level of inflammatory cytokines suggested that ZN could influence not only the intra-cellular inflammatory level but also the inter-cellular inflammatory level. This phenomenon consisted of the previous study that ZN can enhance Akt-mediated IL-10 production and exert anti-inflammatory effects in myeloid cells [41].

Importantly, we noticed the restoration of intercellular tight junction protein (VE-cad and Occludin), indicating the binding force between HUVECs recovered after the treatment of BMP-2 [42]. Haidari *et al.* found that after the treatment of Atorvastatin, VE-cad proteins were retrieved by inhibiting its phosphorylation, and the integrity of endothelial adherens junctions was preserved [43]. Meanwhile, phosphorylation of VE-cad was down-regulated, suggesting inhibition of the internalization of VE-cad. As a result, the permeability of the monolayer of HUVECs was protected. Moreover, the dextran assay (Transwell-experiment with dextran) is typically utilized to evaluate the permeability of cells [44,45]. In our work, we performed the dextran assay in each experiment group. We found that ZN could reduce the amount of dextran that passed through HUVECs and relive the hyperpermeability caused by BMP-2. This result further confirmed the positive effects of ZN.

On the other hand, there were also several flaws in the present study. One major flaw is that we only utilized the *in vitro* cell model to discover the mechanism of BMP-2 and ZN, rather than coupled with a more convincing *in vivo* animal model. Second, since ZN showed potential in treating BMP-2-related side effects, what was the influence of ZN on other aspects of HUVECs, as well as on different cell lines, remained to be discovered.

## Conclusion

**Figure 8:**
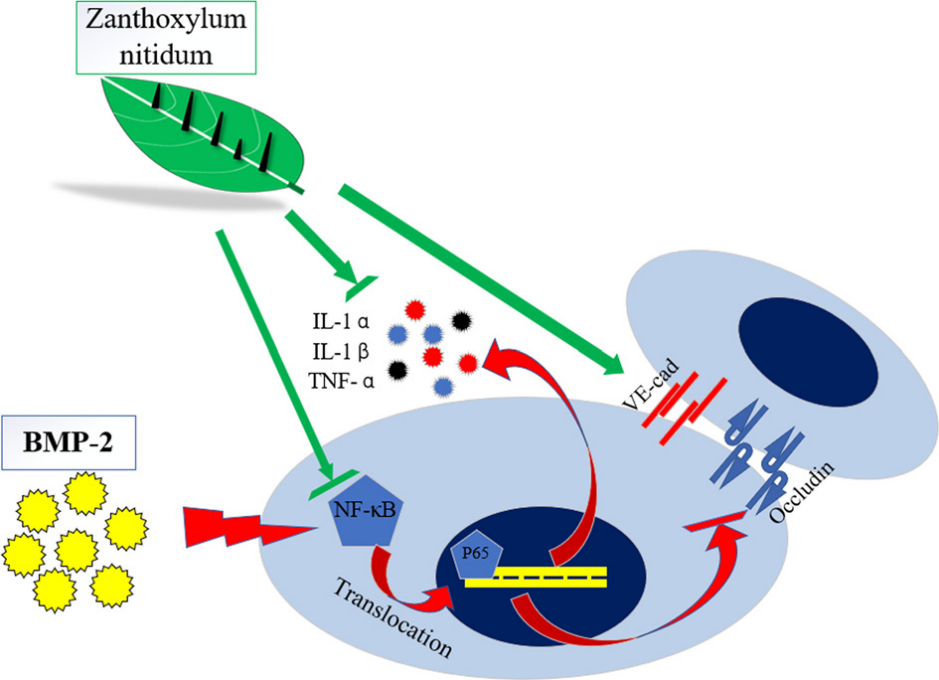
Schematic representation of the mechanism by which ZN down-regulates BMP-2-induced inflammation and hyperpermeability in HUVECs BMP-2 induces inflammatory factors production that activates NF-κB pathway signaling and reduces expression of VE-cad and Occludin. ZN inhibits the BMP-2-induced inflammatory factors production, NF-κB pathway signaling activation, and reduction of VE-cad/Occludin expression.

Above all, for the first time, the present study demonstrates that ZN exerted protective and therapeutic effects against BMP-2-induced inflammation and hyperpermeability with the inhibition of the NF-κB signaling pathway in a concentration-dependent manner (Figure 8). This finding demonstrated that ZN may be a valuable therapeutic candidate against BMP-2-induced side effects during spinal fusion.

## Abbreviations

BMP-2: bone morphogenetic protein-2
ECM: endothelial cell medium
HUVEC: human umbilical vein endothelial cell
IL: interleukin
NELL-1: the osteoinductive growth factor Nel-like protein 1
rhBMP-2: the recombinant human bone morphogenetic protein-2
TNF-α: tumor necrosis factor-α
ZN: Zanthoxylum nitidum
